# A comparative study of CAD/CAM fabricated polyether ether ketone and fiber-glass reinforcement composites versus metal lingual retainers under vertical load (an in vitro study)

**DOI:** 10.1186/s12903-023-03268-5

**Published:** 2023-08-21

**Authors:** Abdullah A. Alabbadi, Essam M. Abdalla, Seham A. Hanafy, Tarek N. Yousry

**Affiliations:** 1 Department of Orthodontics, Faculty of Dentistry, Alexandria University, Champolion street, P. O. Box: 21521, Azarita, Alexandria Egypt; 2 Department of Dental Biomaterials, Faculty of Dentistry, Alexandria University, Alexandria, Egypt

**Keywords:** Fixed retainer, CAD\CAM, Digital orthodontics, FRC, PEEK

## Abstract

**Background:**

Retainer is a necessary procedure when orthodontic treatment complete to avoid relapse due to periodontal fiber elasticity and to allow for alveolar bone regeneration. Compare the influence of vertical force on the failure of three fixed retainers: CAD/CAM polyether ether ketone (PEEK), CAD/CAM fiber glass reinforced composites (FRCs), and lingual retainer wire “Bond-A-Braid™”.

**Materials and methods:**

One hundred and eight maxillary first premolars teeth were randomly allocated to three groups: Group A (CAD/CAM PEEK), Group B (CAD/CAM FRC), and Group C (lingual retainer wire " Bond-A-Braid™”). These retainers were bonded using Assure Plus Bonding Resin and GO TO Paste. For each specimen, a loading cycling and thermocycling machine was used. The failure debonding forces were measured on the interproximal segments using a universal testing machine with a cross-head speed of 1 mm/min. The adhesive remnant index (ARI) was calculated after identifying types of failure with a stereomicroscope at (X 20) magnification.

**Results:**

Group B and group C showed the highest failure bonding forces, with a mean of 209.67 ± 16.15 and 86.81 ± 4.59 N, respectively. However, Group A had a statistically significant lower bond failure force, with a mean value of 45.73 ± 4.48 N. At baseline, there was a statistically significant difference in connector retainer displacement between the three studied groups (p < .001). The ARI score was not statistically significant (p < .001) between the three study groups; for groups A and B, the ARI was predominantly score 3, and group C showed a mixed score of 2 and 3. The failure mode of retainers was investigated using an optical stereomicroscope. In group B, there was a cohesive breakdown in the retainer, and groups A and C exhibited failures primarily in the adhesive at the retainer interface.

**Conclusion:**

All groups differed significantly, with group A having the lowest debonding force and group B having the highest. Furthermore, there was not a substantial variation in ARI, but there was a significant difference in connector retainer displacement and the types of failure amongst the three groups.

## Background

Retention is an important step following orthodontic treatment to prevent relapse. Long-term research found that fixed lingual retainers were efficient at keeping lower incisors in their new position after orthodontic treatment [1].

Various types of retainers have been reported, including those made with wires of various material characteristics and diameters [2], or with fiber reinforcement [3]. The use of computer-aided design/computer-aided manufacturing (CAD/CAM) technology in dentistry for the fabrication of fixed lingual retainers is limited, as research in this area shows. According to the previous studies, CAD/CAM technology was used to fabricate a custom fixed retainer from a block of nickel-titanium, [4] zirconium, [5] polyether ether ketone (PEEK), [6], and fiber glass reinforced composites (FRCs) [7].

In dentistry, PEEK is gaining popularity as an alternative to metal alloys [8]. Even at higher temperatures, PEEK possesses good chemical and mechanical resistance qualities [9]. It is extremely resistant to biodegradation and degradation in organic and aqueous conditions and has a high tensile strength. In this context, one disadvantage of PEEK in dentistry is low wettability, low surface energy and resistance to surface modification by different chemical treatments [10, 11]. This material creates a high-strength appliance for passive retention as a fixed retainer for anterior teeth [12]. A new type of retainer may now be digitally generated from PEEK material and bonded to teeth using CAD/CAM technology to provide a strong, long-lasting, flexible, biocompatible, and more anatomically fitted retainer [12].

Fiber reinforced composites (FRCs) have been used in dentistry for more than 40 years. According to several research, (FRCs) have higher deflection values than traditional stainless steel and metallic wires [13, 14]. There are several applications for new CAD/CAM (FRCs) as a material in dentistry. The CAD/CAM (FRCs) has a number of benefits, including a low specific weight, durability and resilience, lack of firing requirements, special mechanical properties with high flexural strength, physiologically perfect elastic modulus, excellent bond strength when bonded to dental veneering composite. Additionally, it can be appropriate for people who are allergic to metals or who are sensitive to the metallic taste. Less plaque retention and longer-lasting color stability are a result of the retainer’s high degree of polish ability [7, 15].

A study compared the various types wire retainer including flat-braided wire (Bond-A- Braid®, Reliance Orthodontic Products), twisted wire (Ortho Technology, Tampa, Florida), coaxial wire (Ortho Technology, Tampa, Florida), and lingual retainer wire (Ortho-Flex Tech®, Reliance Orthodontic Products, Itasca, IL). They found that there was no superiority over each other [16]. Corresponding to this, Elsorogy et al., reported the highest values of debonding forces were for the Bond-A-Braid, dead soft wire “RESPOND” and FRCs groups, respectively [17].

A research assessed PEEK lingual retainer pads with and without holes to evaluate the optimization of the design of PEEK retainers. The shear bond strength (SBS) of the PEEK pad with a hole was substantially greater than that of the PEEK pad without a hole. While the SBS of the larger pad design was only marginally higher than that of the smaller pads, on the other hand, the 1.5 mm height connector had a substantially lower SBS than the 2 mm [6].

A case report study used a splint fabricated from CAD/CAM glass fiber-reinforced composite, after periodontal treatment procedure, to stabilize the anterior teeth and help in dispersing occlusal stresses, reestablishing functional occlusion, and providing functional comfort when chewing [7, 18].

Since no specific form of retainer can be deemed to be the ideal retainer based on the available evidence, [19] the aim of the study is to compare the debonding force, connector retainer displacement, failure site and adhesive remnant index of three different kinds of lingual retainer: CAD/CAM PEEK, CAD/CAM FRCs, lingual retainer wire " Bond-A-Braid™” fixed retainer.

The null hypothesis is the effect of vertical forces on failure of retainers will not be significantly different between the CAD/CAM (FRCs), (PEEK) and lingual retainer wire (Bond-A-Braid) fixed retainer.

## Materials and methods

### Study design

**Fig. 1:**
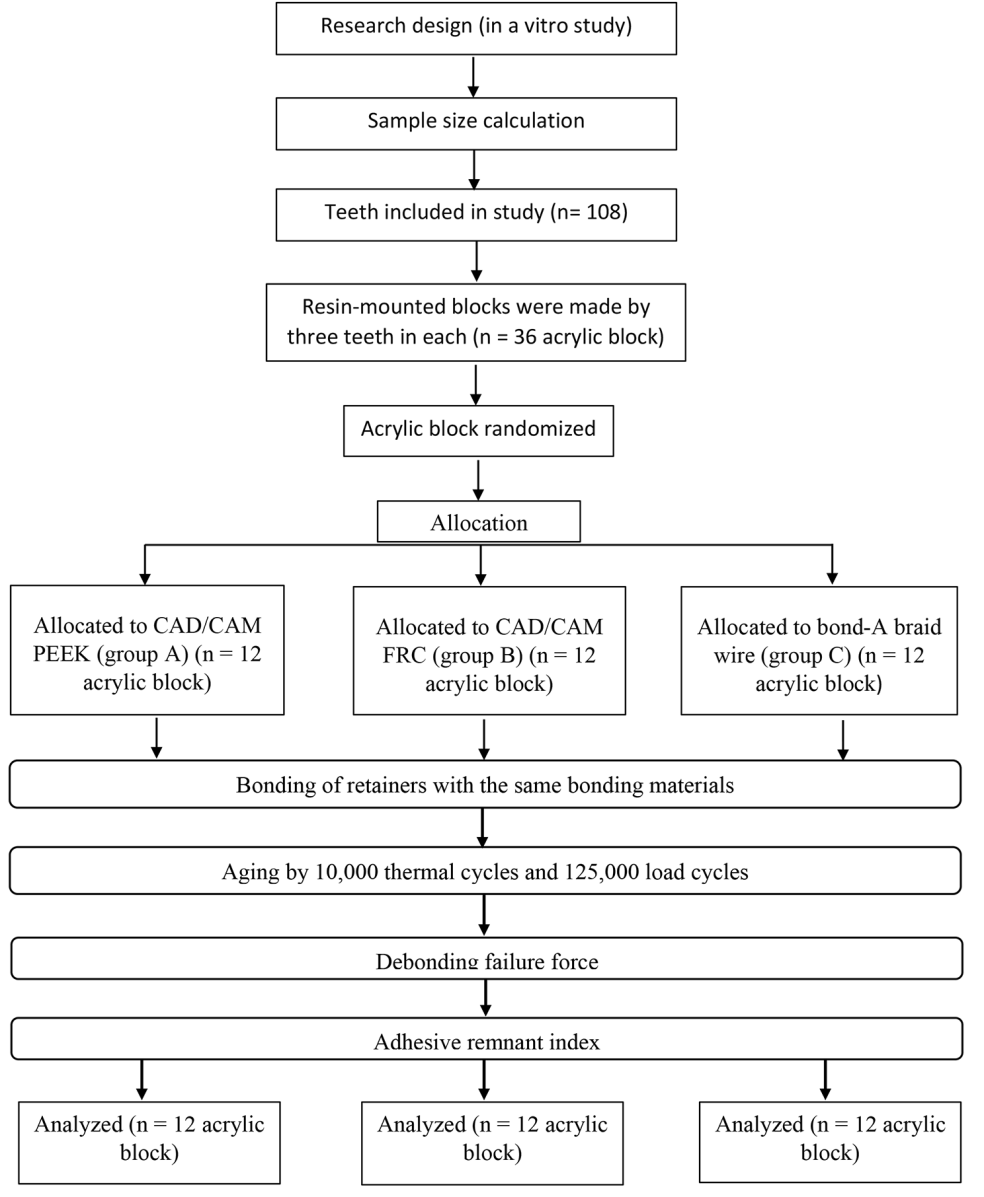
A flowchart shows the study design

This in vitro study was conducted to compare the influence of vertical force on the failure of three different bonded retainers: CAD/CAM (PEEK), CAD/CAM (FRCs), and lingual retainer wire “Bond A Braid” (Fig. 1). All retainers were bonded with Assure PLUS All Surface Bonding Resin and GO TO Paste.

The research ethics committee of the Faculty of Dentistry, Alexandria University (IRB:00010556–IORG:0008839), reviewed and approved the study. All procedures were carried out in accordance with the CRIS regulations and recommendations. The entire study was hosted by Alexandria University’s Orthodontic and Biomaterial Departments.

### Sample size calculation

Based on Elsorogy et al. (2019) [17], adopting a power of 80% (β = 0.20) to detect the force required to fail a fixed retainer (primary outcome) of 0.555, and level of significance 5% (α error accepted α = 0.05), the minimum required sample size was found to be 12 specimens per group (number of groups = 3) (Total sample size = 36 specimens) [20]. Any sample that withdrew from the study for any reason was replaced in order to maintain the sample size [21]. The sample size was calculated using GPower software (v. 3.1.9.2) [22].

### Procedures

#### Sample grouping and preparation

The study included 36 specimens divided equally into three groups: Group A (CAD/CAM Polyether ether ketone (PEEK) retainer), Group B (CAD/CAM fiber-reinforced composite (FRC) retainer) and Group C (Flat braided wire ― Bond A. Braid).

One hundred and eight freshly extracted upper premolars for orthodontic treatment purposes were gathered. Each individual signed an informed consent form to enable the use of their premolars. If the individual was younger than 18 years, a guardian signed the permission on their behalf. The teeth had to be uncracked, caries-free, and decalcified free in order to be used in the current investigation. They were examined by a magnifying glass lens.

The teeth were cleaned with water, then pumiced and kept in distilled water. At the start of the experiment, each of the three teeth was assigned an identification number ranging from 1 to 36 and kept in a separate, marked lab bottle filled with distilled water that was replaced every week. Teeth were allocated into three trial groups using a random number generator. Each of three premolar teeth harmonized to generate a contact area to simulate the intraoral position of lower anterior teeth; The premolars’ proximal surfaces were reduced in width to 6 mm to mimic the lower central incisors’ mesiodistal width by using a dental disc. The roots were soaked in molten wax 2 mm below the cemento-enamel junction (CEJ) to make a 0.5 to 1 mm thick wax layer.

To guarantee that the roots of each of the three teeth are parallel, Roth brackets with a “0.022 slot” are bonded to the buccal surface of the teeth. A rectangular 0.021*0.025-inch stainless steel wire is passively attached to the brackets using O-ties. In order to maintain a consistent thickness of the elastomeric material surrounding the root, each of the three teeth was maintained perpendicular to the mold’s base and parallel to each other using a specially milled metal holder that was attached to the surveyor. The manufacturer’s drop technique was used to pour the chemically cured acrylic resin into the metal holder [23].

**Fig. 2:**
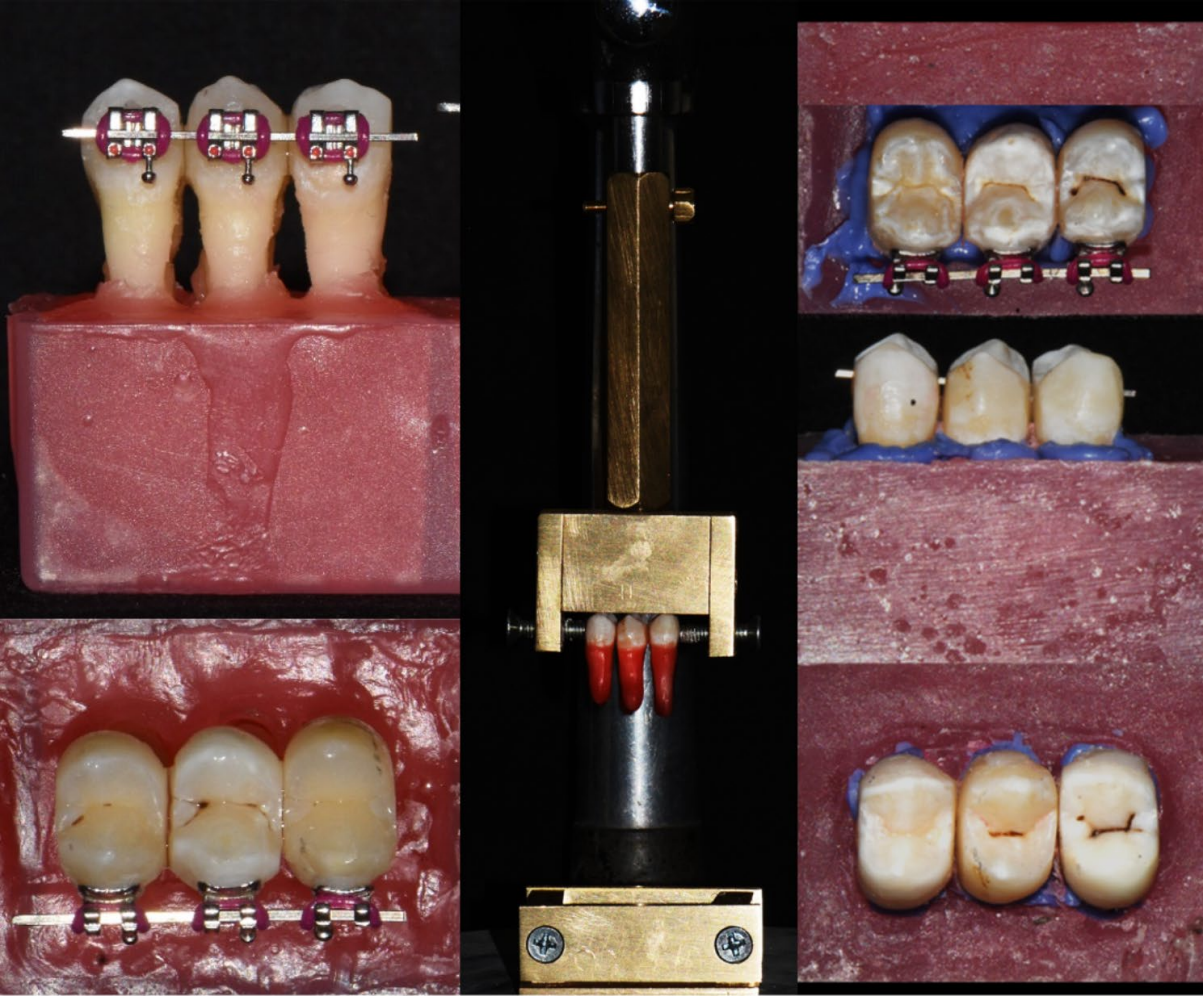
A sample of the teeth preparation used for bonded fixed retainers

After the acrylic had fully polymerized, the wax was removed from the root surface and resin cylinder (or “alveolus”) using hot water to prevent wax remnants from adhering to the teeth’s lingual surface and interfering with the bonding procedure. Following that, the teeth were removed from the acrylic block. In order to mimic periodontal ligaments and normal tooth movement, the socket’s root in the acrylic block was filled with the elastomeric material, the teeth were reinserted (Zhermack Zetaplus C Silicone Intro Kit Catalyst + Light Body), and the brackets were debonded from the labial surface (Fig. 2).

#### Fabrication of CAD/CAM retainer

**Fig. 3:**
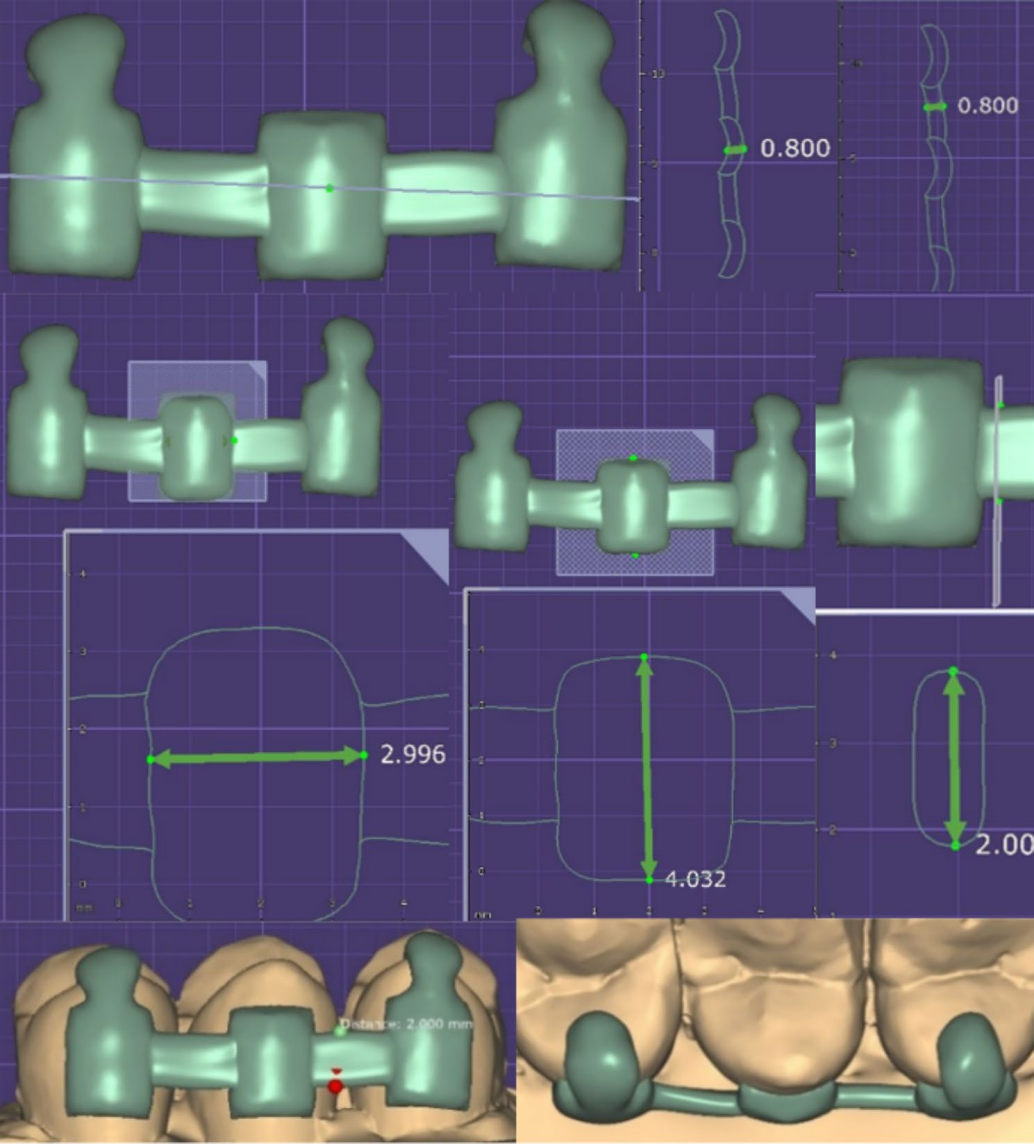
A Digital workflow design of CAD CAM PEEK and FRCs retainer

In order to create virtual PEEK and FRCs retainers, the tooth model was scanned using an intraoral dental scanner (Omnicam scanner, Dentsply Sirona) to create a virtual model file. The retainers were designed using a software system (Exocad Dental CAD 2.2 Valletta). The retainers had pad on each tooth with 3 mm width and 4 mm height, and a connector with 2 mm height and 0.8 mm thickness. Each retainer had occlusal guides on the occlusal surfaces of the teeth, which allowed for simpler and more accurate placement of the retainer (Fig. 3). After the design of the retainer was finished, the Roland DWX-52D milling machine was loaded with “White” DD-PEEK-MED blocks (DentalDirekt, Spenge, Germany), and FRCs (Trilor®, BiolorenS.r.I.) blanks to create the final CAD/CAM PEEK and FRCs retainer. Each retention pad had a hole hand-drilled into the center with a 1 mm blue round diamond bur.

#### Bonding procedure of retainer (Fig. 4)

**Fig. 4:**
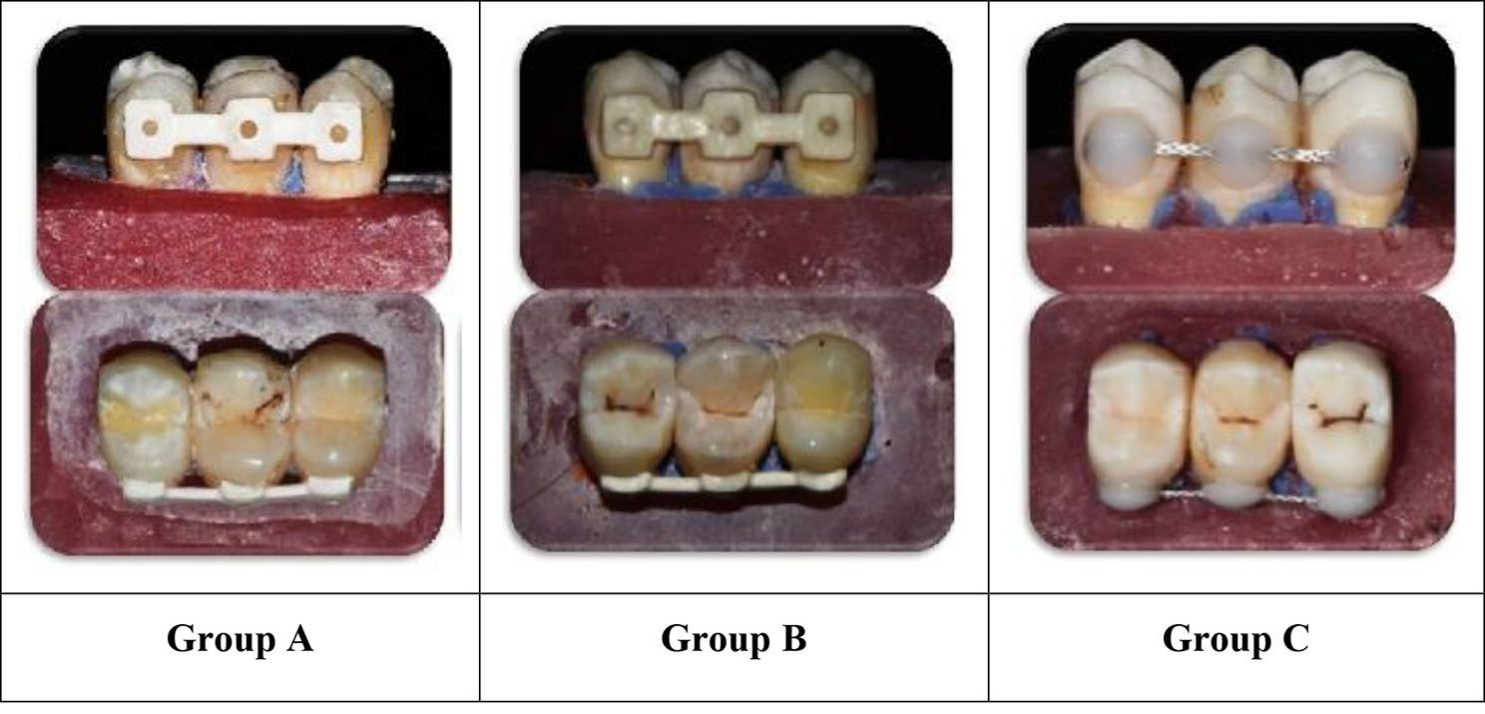
Bonding sample of groups A, B and C

The same operator made and bonded all of the retainers in the study, following the manufacturing sequence shown below.

For uniformity the lingual surface of each premolar was cleaned with oil-free pumice for 20 s using a prophylaxis brush, then rinsed and dried. The enamel surfaces were etched for 30 s with 37% phosphoric acid (Meta Biomed), rinsed with distilled water, and dried for 20 s until the chalky white enamel appeared. After that, the Assure Plus bonding resin (Reliance Orthodontic Products, USA) was applied uniformly with a micro brush, gently air-blown onto the enamel surface, and photopolymerized for 10 s on each tooth surface (Woodpecker i-led, 2300 mW/cm, woodpecker, china).

The CADCAM FRCs and PEEK retainers in Groups A and B were washed with distilled water for 60 s and air dried. After that, applying a thin coating of Assure Plus bonding resin for 20 s, followed by a gentle air stream for 5 s, and light curing for 10 s. The GO TO Paste (Reliance Orthodontic Products, USA) was then applied to the retainer and then place on the enamel surface and pressed with equal force. Following that, carefully removing any excess, the teeth were light-cured for 40 s in all directions. The occlusal guide was removed by a thin wheel diamond bur after complete retainer bonding.

To standardize the amount of composite in Group C, a dome-shaped mini mold (Ormco.pl) was used for each bond. The GO TO paste was applied to the metal retainer, the mold was filled with it and pushed against the metal retainer. Before curing for 40 s, excess composite was scraped from the mold’s borders. The composite mass on each tooth is 4 mm in diameter and 1.5 mm in depth.

#### Aging procedure

**Fig. 5:**
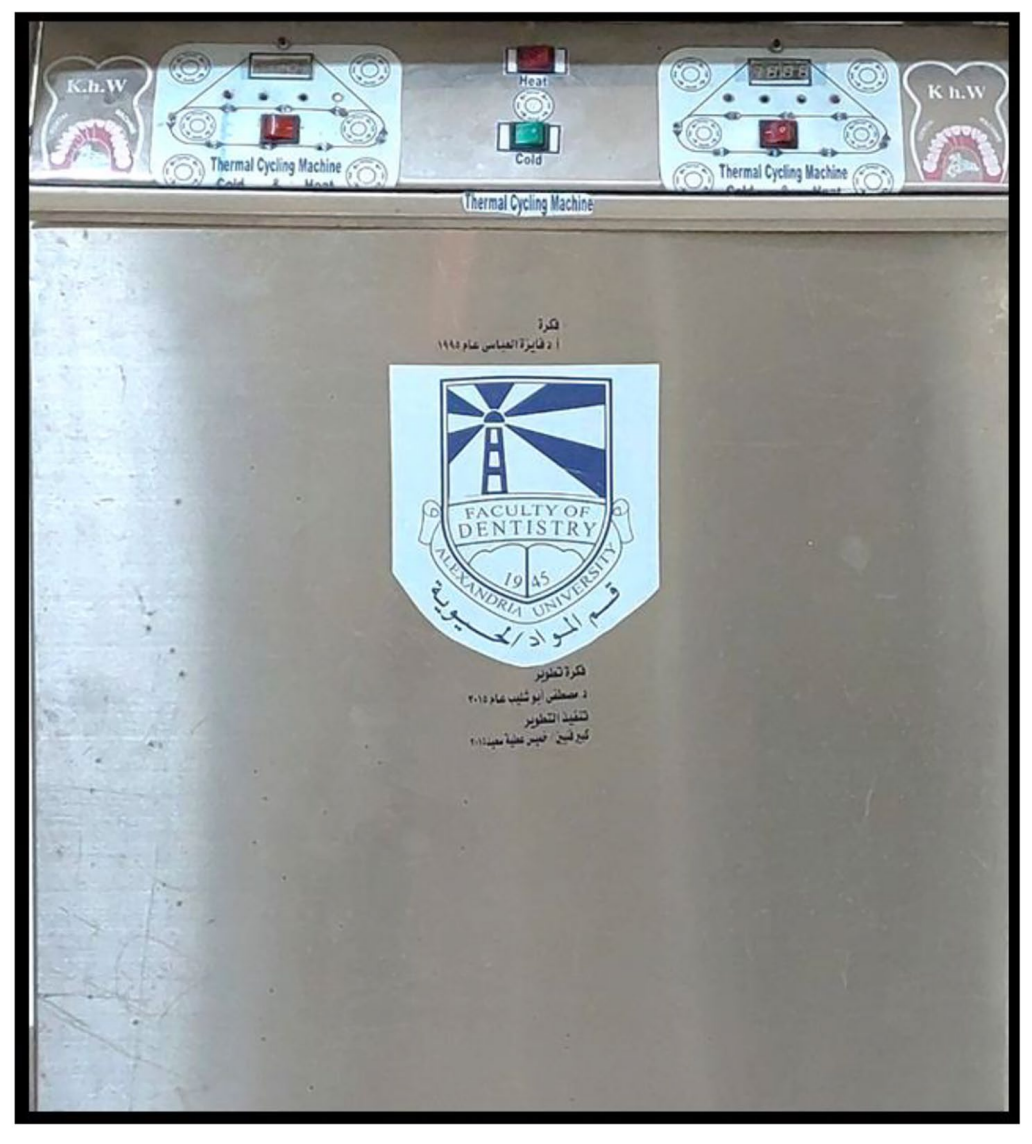
Thermocycling is applied by the thermocycling machine

**Fig. 6:**
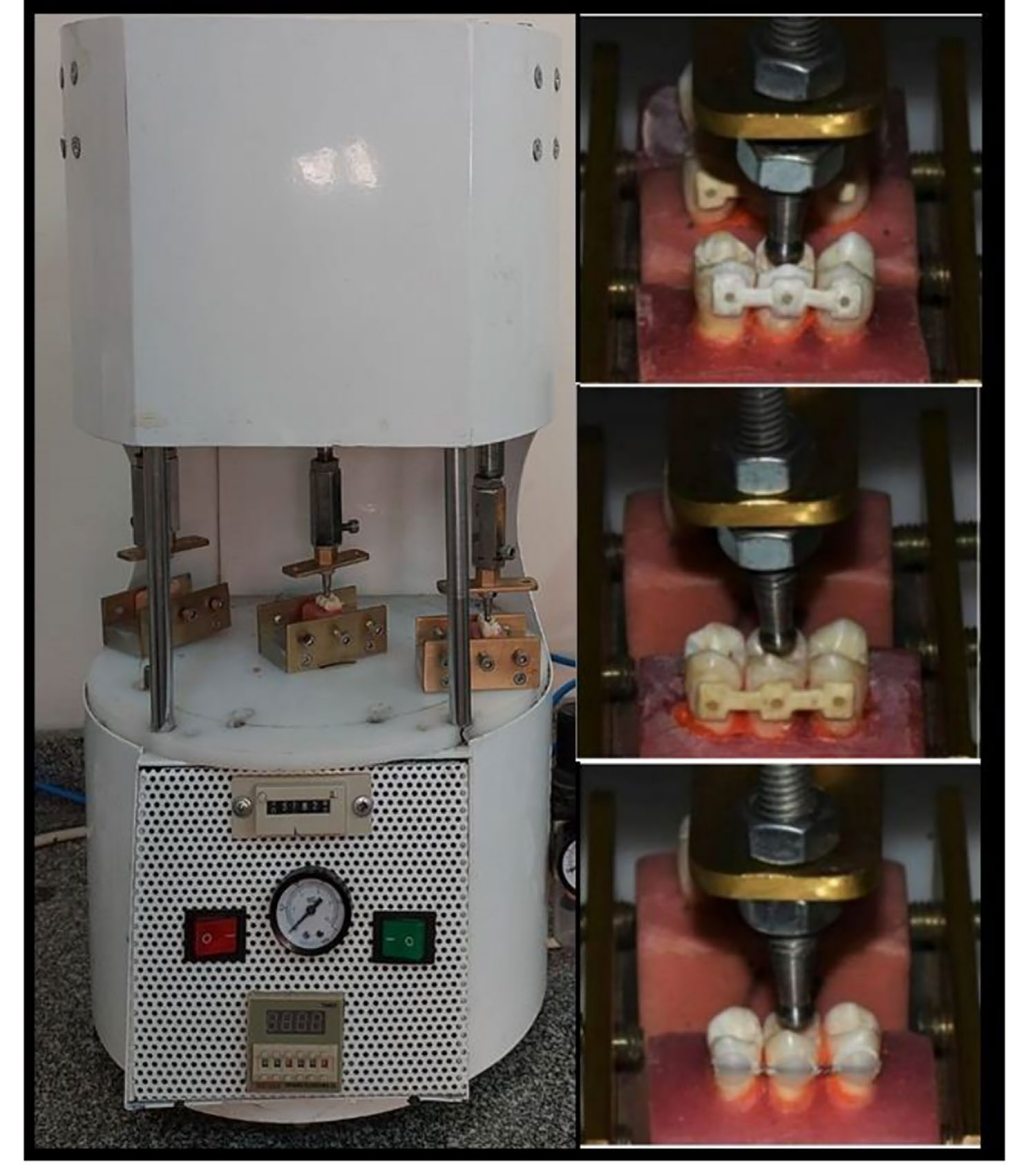
The loading cycling machine applied a load on the occlusal of the middle tooth

To replicate around six months in the oral environment, all of the samples were thermocycled 10,000 times in water between 5 and 55 degrees Celsius using a thermocycling machine with dwell times of 60 s and transition times of 15 s (Fig. 5) [17, 24]. In order to simulate the complex intraoral mechanical loading conditions. The same specimens were mounted in a loading cycling machine and subjected to an intermittent force of 40 N with a short stroke distance of 10 mm in the center of the specimen’s middle premolar tooth for 125,000 loaded cycles to replicate six months of clinical service at a loading frequency of 1 Hz (Fig. 6) [17, 25]. Any debonding during the aging process was recorded, including the failure site and type.

#### Failure bonding force

**Fig. 7:**
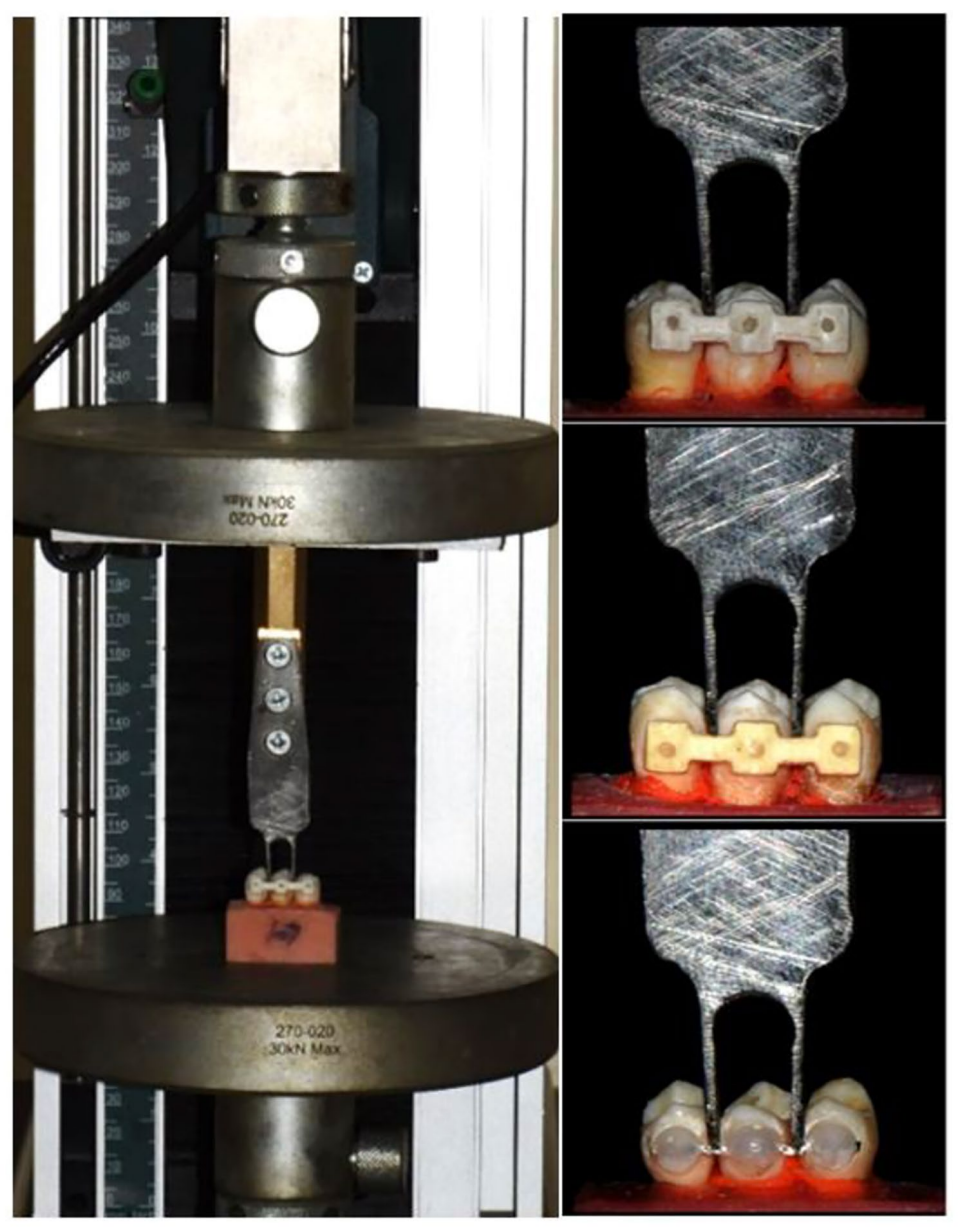
Force applied by the universal testing machine using a fork at the center of the retainer of the interdental segments between the teeth

The crosshead speed was set to 1 mm/min when the samples were loaded into the universal testing machine (5ST, Tinius Olsen England 2018). The initial debonding force was measured in Newtons with a crosshead positioned in the middle of connector retainer (Fig. 7) [26].

### Connector retainer displacement

The same universal testing machine was used to determine the maximum connector retainer displacement that existed prior to failure (5ST, Tinius Olsen England 2018). The displacement was measured in millimeters.

### Adhesive remnant index

After debonding, the remaining adhesive on the enamel surface was noted using an optical stereomicroscope at 20X magnification (Olympus SZ-CTV, Japan). The adhesive remnant index (ARI), which considered how much adhesive was remained on the retainer, was used to identify and grade the failures on a scale of 0 to 3. When more than one tooth fails, the average of the adhesive remnants should be calculated [27].

- Score 0: No adhesive remained on enamel.
- Score 1: Less than 50% of the adhesive remained on enamel.
- Score 2: More than 50% of the adhesive remained on enamel.
- Score 3: All adhesive remained on enamel.

In order to identify the bond failure site, which could have been the retainer itself, the composite-retainer interface, or the composite-enamel interface, retainers were examined under an optical stereomicroscope at a magnification of x20 [28].

For dependability, the same examiner repeated the score calibration after two weeks. After calibrating the ARI assessment, the kappa statistic was determined (K = 0.79), suggesting extremely excellent intra-examiner reliability [29].

### Statistical methodology

- Data were collected and analyzed using Statistical Package for Social Science (SPSS) program for statistical analysis (ver 25) [30].
- Kolmogorov-Smirnov test of normality revealed no significance in the distribution of the variables, so the parametric statistics was adopted [31].
- Data were described using minimum, maximum, mean, standard deviation, 95% CI of the mean and 25th to 75th percentile.
- Categorical variables were described using frequency and percentage.
- Comparisons were carried out between more than two independent normally distributed subgroups using one-way ANalysis Of VAriance (ANOVA) test [32]. When F ratio of ANOVA was significant Levene test of homogeneity of variances was done, and if significant Brown-Forsythe Robust test was adopted. Post-hoc multiple comparisons [33] was done Games-Howell method [34].
- Chi-square test was used to test association between qualitative variables [35]. Monte Carlo corrections [36] was carried out when indicated (n x m table and > 25% of expected cells were less than 5).
- During sample size calculation, beta error accepted up to 20% with a power of study of 80%. An alpha level was set to 5% with a significance level of 95%. Statistical significance was tested at *p* value < 0.05 [37].

## Results

**Fig. 8:**
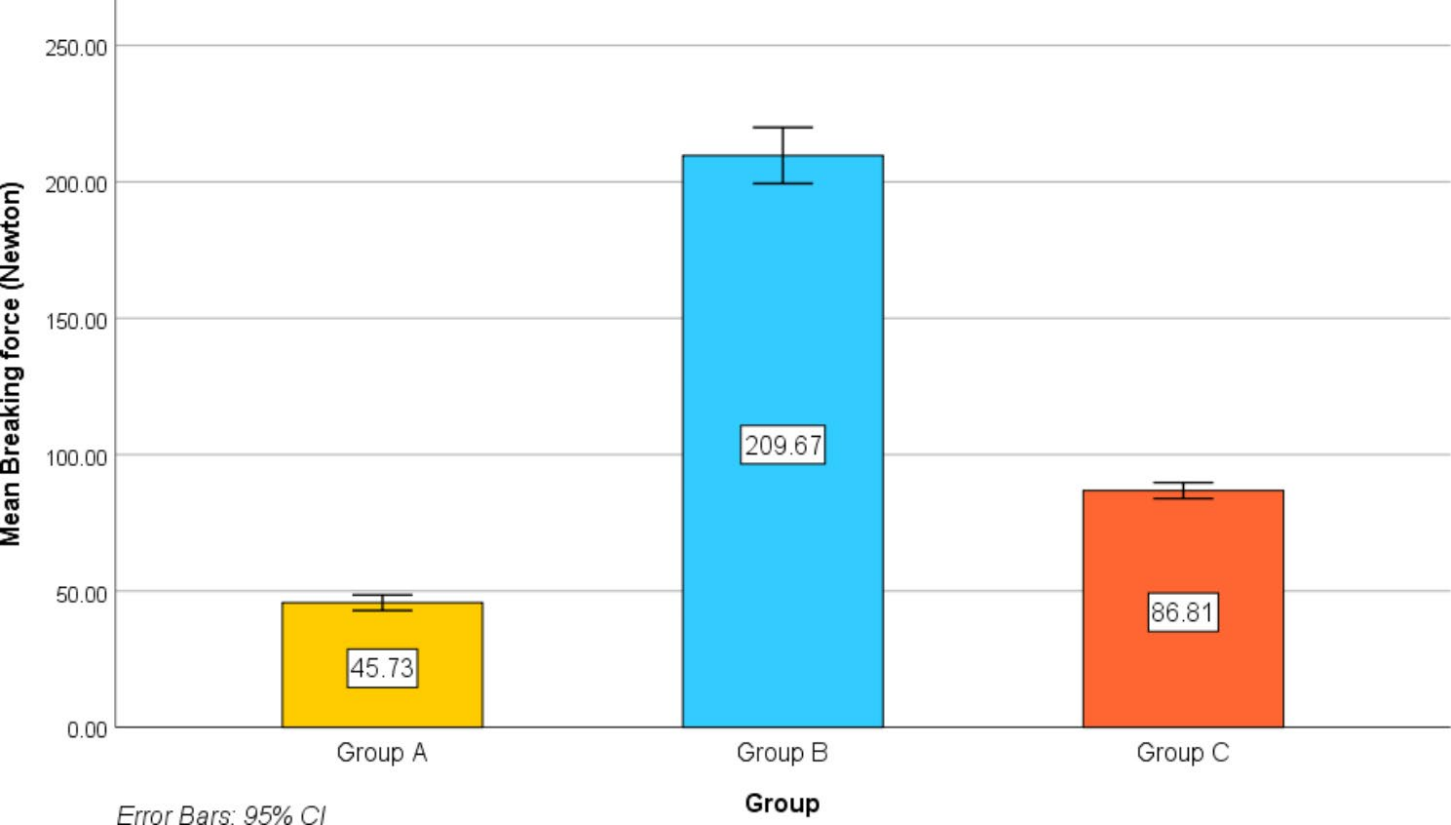
Simple bar chart for the mean debonding force (Newton) of the studied groups

For any of the samples, there were no retainer failures throughout the thermal and loading cycles. The mean debonding forces were 45.73 ± 4.48, 209.67 ± 16.15 and 86.81 ± 4.59 N in group A, B and C respectively. There was a statistically significant difference in debonding force among the three studied groups at baseline (*p* < .001) (Table 1; Fig. 8).

**Table 1:** Bonded retainer failure forces Mean ± Std. Deviation (in Newton) and comparison of three study groups

	Group			Test of significance *p* value
	Group A (n = 12)	Group B (n = 12)	Group C (n = 12)	
Breaking force (Newton)				
-Min-Max -Mean ± Std. Deviation -95% CI for mean	40.10–54.20 45.73 ± 4.48 42.88–48.57	179.00-231.00 209.67 ± 16.15 199.41-219.93	76.10–92.90 86.81 ± 4.59 83.89–89.72	F_(BF)_ = 867.620 *p* < .001*
	Pairwise comparison using Games-Howell			
	**Group A**	**Group B**	**Group C**	
**Group A**		Diff=-163.94167 *p* < .001*	Diff=-41.08333 *p* < .001*	
**Group B**			Diff = 122.85833 *p* < .001*	
**Group C**				

n : Number of samples.

Min-Max: Minimum – Maximum.

CI: Confidence interval.

* : Statistically significant (*p* < .05).

NS: Statistically not significant (*p* ≥ .05).

Pairwise comparisons revealed that the debonding force was statistically significantly higher in the group B compared with the group A and group C (*p* < .001, *p* < .001 respectively). Group C was statistically significantly higher compared to the group A (*p* < .001).

In groups A, B, and C, the mean connector retainer displacement was 0.13 ± 0.07 mm, 0.73 ± 0.13 mm, and 1.33 ± 0.30, respectively. There was a statistically significant difference in connector retainer displacement amongst the three investigated groups (*p* < .001) Table 2.

**Table 2:** The connector retainer displacement an among the three groups in mm

	Group			Test of significance *p* value
	Group A (n = 12)	Group B	Group C (n = 12)	
Retainer displacement (mm)				
-Min-Max -Mean ± Std. Deviation -95% CI for mean	0.05–0.30 0.13 ± 0.07 0.08–0.17	0.56–0.95 0.73 ± 0.13 0.64–0.81	0.89–1.93 1.33 ± 0.30 1.15–1.52	F_(BF)_ = 119.873 *p* < .001*
	Pairwise comparison using Games-Howell			
	**PEEK retainer**	**FRC retainer**	**Metal retainer**	
**PEEK retainer**		Diff=-0.59833 *p* < .001*	Diff=-1.20833 *p* < .001*	
**FRC retainer**			Diff=-0.61000 *p* < .001*	
**Metal retainer**				

n : Number of patients

Min-Max: Minimum – Maximum

CI: Confidence interval

* : Statistically significant (*p* < .05)

NS: Statistically not significant (*p* ≥ .05)

Pairwise comparisons revealed that the retainer displacement was statistically significantly lower in group A compared with the group B and group C (*p* < .001, *p* < .001 respectively). Group C was statistically significantly higher compared to the group B (*p* < .001).

**Fig. 9:**
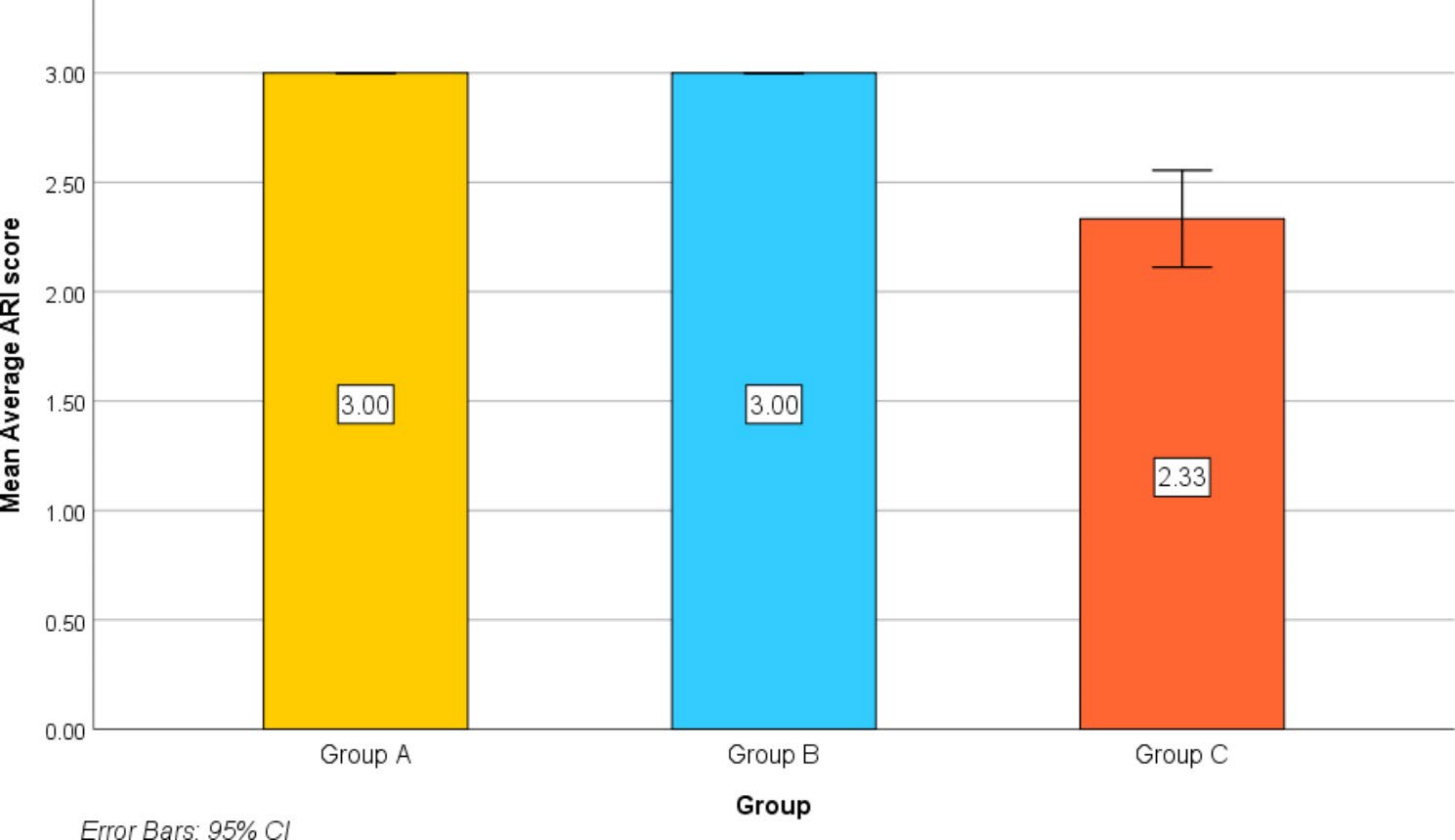
The average of adhesive remnant index (ARI) scores for the three study groups

Table 3 as well as Fig. 9 are described the average ARI score among the three studied groups. In group A and B, the mean of ARI score was 3.00 ± 0.00, while in group C the mean of ARI was 2.33 ± 0.35. Pairwise comparisons revealed that the average ARI score was statistically significantly lower in the metal retainer group compared with the PEEK retainer groups and FRC retainer group (*p* < .001, *p* < .001 respectively).

**Table 3:** The average adhesive remnant index (ARI) for each of the three study groups

	Group			Test of significance *p* value
	**Group A** (n = 12)	**Group B** (n = 12)	**Group C** (n = 12)	
**Average ARI score**				
-Min-Max -Mean ± Std. Deviation -95% CI for mean	3.00–3.00 3.00 ± 0.00 3.00–3.00	3.00–3.00 3.00 ± 0.00 3.00–3.00	1.67–2.67 2.33 ± 0.35 2.11–2.55	F_(BF)_ = 44.000 *p* < .001*
	Pairwise comparison using Games-Howell			
	**Group A**	**Group B**	**Group C**	
**Group A**		Diff=-0.000	Diff = 0.66667 *p* < .001*	
**Group B**			Diff = 0.66667 *p* < .001*	
**Group C**				

n : Number of patients

Min-Max: Minimum – Maximum

CI: Confidence interval

* : Statistically significant (*p* < .05)

NS: Statistically not significant (*p* ≥ .05)

NA : non applicable (due to exact match)

**Fig. 10:**
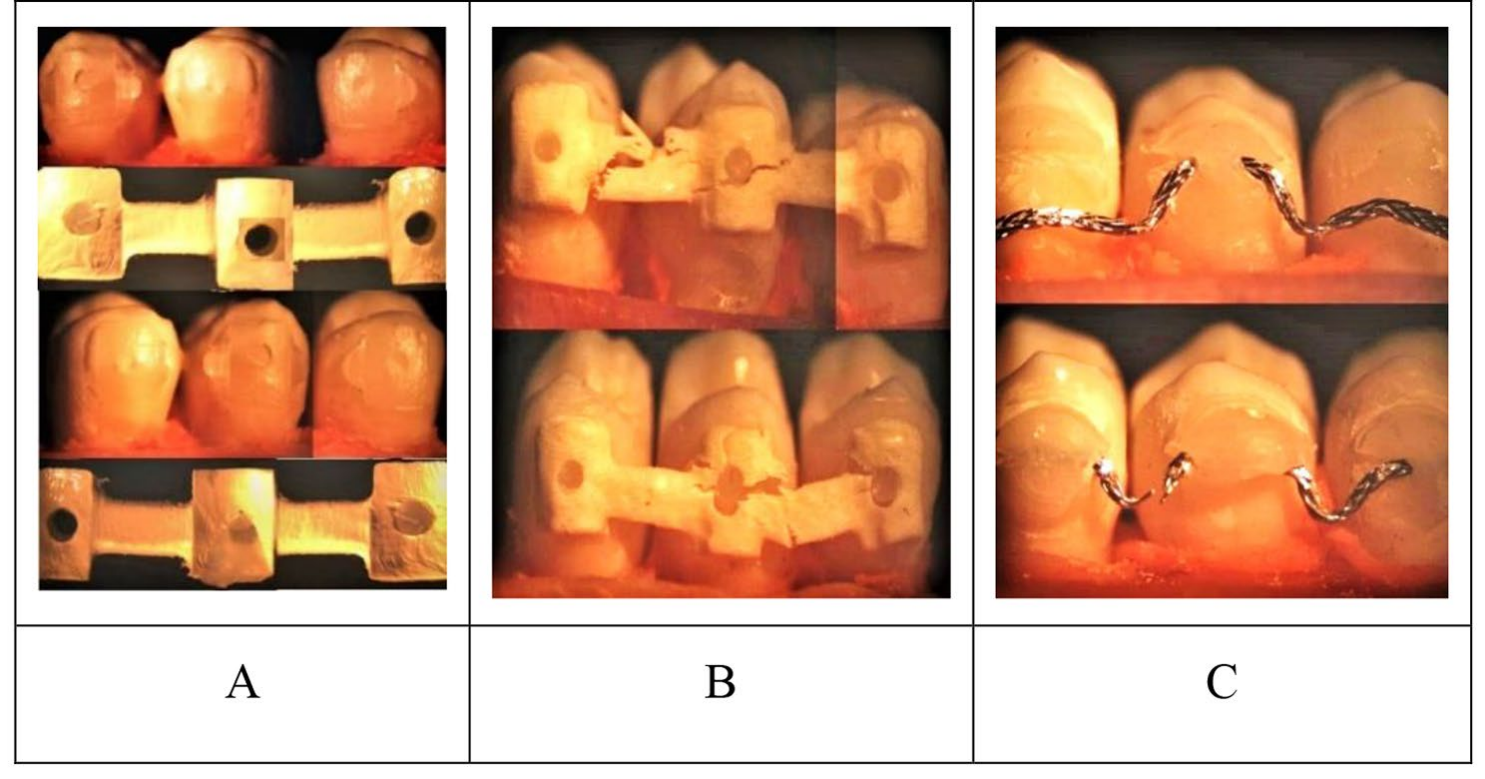
**(A)** The ARI for group A under the optical stereomicroscope displays a score of 3 and an adhesive failure site at the retainer interface. **(B)**. Group B displays a score of 3 and a cohesive failure pattern in the retainer. **(C)** Group C is displaying scores of 2 and 2 as well as a failure pattern that combines failure of the cohesive retainer and adhesive at the retainer interface

Table 4; Fig. 10 illustrates the failure type of retainers under an optical stereomicroscope. There is a statistically significant difference in the occurrence of adhesive at the retainer interface failure mode (*p* < .001) and Cohesive in retainer failure mode (*p* < .001) among the three studied groups. In group A all (100%) of the samples showed failure that is adhesive at retainer interface (AR), in group B all (100%) of the samples showed failure that is cohesive in retainer failure (CR), finally in group C 75% of samples showed failure that is adhesive at retainer interface and 25% of samples showed failure that is cohesive in retainer.

**Table 4:** Counts and percentages of different modes of failure retainers in the study groups

Failure mode test	Group			Test of significance *p* value
	Group A	Group B	Group C	
Cohesive in the tooth	0 (0.00%)	0 (0.00%)	0 (0.00%)	NA
Adhesive at the tooth interface	0 (0.00%)	0 (0.00%)	0 (0.00%)	NA
Cohesive in resin	0 (0.00%)	0 (0.00%)	0 (0.00%)	NA
Adhesive at the retainer interface	12 (100.00%)^**a**^	0 (0.00%)^**b**^	9 (75.00%)^**a**^	c^2^_(df=2)_ = 26.743 *p* < .001*
Cohesive in retainer	0 (0.00%)^**a**^	12 (100.00%)^**b**^	3 (25.00%)^**a**^	c^2^_(df=2)_ = 26.743 *p* < .001*

n : Number of sample.

NA : non-applicable (due to exact match).

## Discussion

Retention after orthodontic treatment is crucial for a variety of reasons, including gingival and periodontal rearrangement, the type of therapy, and growth changes that occur after treatment that contribute to relapse. For these reasons, it is commonly recommended to use a permanent retainer in the long term to preserve treatment outcomes [1, 38]. A previous study revealed that persons with permanent lingual retainers had better teeth alignment than those who did not after 5–10 years of retention [26]. The most significant advantage of use of bonded retainer is that it does not need patient cooperation, as opposed to removable retainers, while the main disadvantage is that these retainers are prone to breaking and failing [38].

The use of CAD/CAM technology in dentistry was initially presented more than 30 years ago [39]. These days, a fixed retainer has been manufactured using CAD–CAM systems. A fixed retainer was formed using various materials and methods by the CAD/CAM technology. A recent paper reported the fabrication of a custom lingual retainer cut from a nickel-titanium, Zirconium, polyether ether ketone (PEEK) and Fiber Reinforced Composite (FRC) blocks with CAD/CAM technology [4–7].

Polyether ether ketone (PEEK) is a polycyclic, aromatic, thermoplastic polymer with a linear structure that is semi-crystalline. Moreover, it possesses suitable mechanical and electrical qualities for dental production and integration, including resistance to high temperatures and hydrolysis [40]. New CAD/CAM fiber-reinforced composite splints have a wide range of indications as a material used for permanent or temporary dental restorations. It combines high flexural strength, possessing a physiologically perfect modulus of elasticity, with excellent bond strength when it is bonded to dental veneering composite [15].

The purpose of this study was to evaluate the vertical load failure, connector retainer displacement, adhesive remnant index (ARI) and failure mode of three different types of fixed retainer; PEEK, FRC and flat-braided wire (Bond-A-Braid) fixed retainer, which were all bonded with GO TO paste and Assure Plus Resin Bonding.

One hundred and eight maxillary first premolars were used in this study, and the teeth were divided into three groups. Lower incisor teeth are the most commonly used fixed lingual retainer teeth. Because of the scarcity of extracted lower incisors, we used premolars teeth and reduced their size to 6 mm in width to mimic the size of lower incisors, and every three teeth were fixed in an acrylic block. Applying elastomeric impression material to the tooth roots while applying force was an excellent method to mimic the flexibility of the periodontium [23]. In CAD/CAM PEEK and FRC retainers, we used Exocad software to design the retainer as three pads in sample with 3 mm in width and 4 mm in height and the connector was 2 mm in height while the thickness of the retainer was 0.8 mm. After milling, we added a hole about 1 mm in diameter in the center of the pad of each retainer to increase its mechanical retention. The mini mold was used to guarantee a consistent amount of composite on the lingual retainer wire (Bond-A-Braid) [41].

Bonded retainers experience cyclic stresses in the oral cavity as a result of mastication, occlusion, and parafunctional behaviors [42, 43]. To mimic intraoral stressors in vitro, thermocycling and loading cycling are frequently used. Moreover, the specimens were subjected to vertical loads in order to evaluate their strength and resistance. In this investigation, using a vertical force on the retainer to imitate a clinical biting condition. Reynolds et al. discovered that a vertical force produces the best bond strength values when compared to a tensile force in either a horizontal or vertical position [44]. However, bond strength is affected not just by the direction of the applied force but also by its position. Several authors have proven that the debonding force values are lowest when force is applied to the interdental region. Therefore, we chose the weakest area to determine the minimum force required for debonding [26].

### Bonding failure

In this study, the teeth underwent 10,000 thermal cycles and 125,000 load cycles with 20 N load to simulate 6 months of clinical service [17]. None of the specimens were damaged in this process. The results showed significant difference in debonding forces of the three groups; thus, the null hypothesis of the study was rejected. This study showed that the CAD/CAM FRC retainer had the highest mean values of debonding forces 209.67 ± 16.15, while the lingual retainer wire (Bond-A-Braid) and CAD/CAM PEEK retainer showed the lowest mean values of debonding forces 86.81 ± 4.59, 45.73 ± 4.48 respectively. In contrast to this study, Riyadh Ruwiaee et al [6]. showed that the CAD/CAM PEEK retainer’s deboning force was 275 N, which exceeded the amount recorded in this study. This difference may be due to the different surface treatment with 98% sulfuric acid for 60 s used to treat the CAD/CAM PEEK retainer surface, which showed an increase in debonding load. Therefore, by modifying the design of a CAD/CAM PEEK retainer to decrease the surface area and use it as a lingual wire retainer. The debonding forces of Bond-A-Braid wire was found to be 86.81 ± 4.59 N which was significantly different from debonding force reported by ElSorogy et al. and A. Golshah et al. 46.27 ± 12.28 N, 55.57 N respectively [16, 17]. Also, the debonding forces of CAD/CAM FRC retainer in the present study was 209.67 ± 16.15 which was higher than the value reported ElSorogy et al. 30.09 ± 15.73 N [17]. This difference could be due to the different retainer materials and adhesive systems, which used the Tetric-N Flow adhesive system, Fiber reinforced composite “Infibra Ribbon” and Transbond XT adhesive (#M Unitek, Monrovia, CA, USA) respectively, in addition to the customized FRC retainer that is more anatomically adapted on the tooth surface.

### Connector retainer displacement

The results indicated that the connector retainer displacement of the Bond-A-Braid wire had the highest mean values, 1.33 ± 0.30 mm, while the CAD/CAM FRC and PEEK retainers showed the lowest mean values of connector retainer displacement, 0.73 ± 0.13 mm, 0.13 ± 0.07 mm respectively. According to the obtained results, the connector retainer displacement of the Bond-A-Braid retainer was 1.33 ± 0.30 mm, which was not significantly different from the value reported by Elsorogy et al [17]. 1.28 ± 0.77 mm. Furthermore, the mean connector retainer displacement of the CAD/CAM FRC retainers in this study was 0.73 ± 0.13 mm, which differed significantly from the values published by Elsorogy et al. 1.34 ± 0.47 mm [17]. This variance may be caused by the use of different adhesives, composite resins, and material types.

### Adhesive remnant index

The adhesive residual index (ARI) is one of the most widely used measures for determining how much adhesive remains on the enamel surface after retainer debonding [45]. The index, on the other hand, reflects the location of the bond fracture after rating each tooth from 0 to 3. If less adhesive remains on the enamel after debonding, the clean-up will be safer, and the risk of enamel injury will be decreased [46]. The findings of this study supported the findings of Cook et al [41]., A. Golshah et al [16]., El-Sorogy et al [17]. and Kotta et al [47]. in that there were no statistically significant differences in the ARI scores of the three groups, with the majority of scores in groups A and B being score 3 and those in group C being either score 2 or 3. In fact, because the most adhesive can still be seen on the enamel surface, the retainer-adhesive interface is regarded to be the optimum way for debonding. As a result, the most favorable mode of failure in debonding retainers is an ARI score of 2 or 3, because it reduces the possibility of enamel damage [47, 48].

When evaluating the responses of the tested retainer materials, it is crucial to take the failure site into consideration. The adhesive retainer interface was the most common point of failure in this study of CAD/CAM PEEK and lingual wire retainers; however, when looking at CAD/CAM FRC retainers, cohesiveness within the retainer was the main point of failure.

This study was conducted as an in-vitro study to have a more standardized bonding protocol, allowing independent evaluation of the debonding force of different materials retainer [49]. It is still unclear how much debonding force is required for oral fixed retainers despite significant research on the bonding force of fixed retainers. Generally speaking, bonded orthodontic attachments must have sufficient adhesion to withstand masticatory forces for the duration of the retention period without failing; nevertheless, the debonding force shouldn’t be too high to prevent substrate loss following debonding [50]. The optimal orthodontic bonded attachment must have bonding forces between 5 and 50 N, even if these values are essentially theoretical [50]. However, lingual retainers are less susceptible to intraoral pressures. As a result, they might also be vulnerable to decreased bond strength levels [44]. In contrast, Cooke et al. thought that this value could not be applied to retainer wires because the vertical forces applied to the retainer are not evenly distributed throughout its length, causing the simultaneous formation of shear, tensile, and shrinkage forces [41]. It should be emphasized that additional factors, such as bonding method and adhesive type, might also contribute to retainer breakage in the oral cavity [51]. Unfortunately, controlling all of these variables was beyond the scope of this study.

The debonding forces of all tested retainers in this research, including the CAD/CAM PEEK, FRC and “Bond-A-Braid™” retainers, exceeded the minimal debonding force required [44]. It may be possible to decrease the debonding force of CAD/CAM FRC by modifying the design of the retainer to decrease the area of adhesive to the tooth surface so that it can be used as a wire retainer.

The reduce stiffness of the multistranded wires is considered an advantage over a rigid CAD/CAM PEEK and FRC retainer in terms of physiologic tooth movement, but the higher resilience and spring back make it unreliable in terms of passivity. Due to the greater stored energy in the multistranded wire during chair side adjustment, low forces may be expressed over longer times. Unwanted tooth movement may occur if these mild forces exceed the periodontal limitations [52].In contrast to CAD/CAM PEEK and FRC retainers, which have greater stiffness and an anatomically adapted passive state. In addition, to reduce the stiffness of the CAD/CAM PEEK and FRC retainers, the design could be modified to be in a wire form [53–55]. The in vitro research cannot accurately replicate the clinical situation, so its results cannot be used right away in the clinical setting. In order to obtain more reliable results, more clinical research will be required.

A limitation of this in vitro study might be represented by the fact that it was conducted on premolars teeth and not on lower incisors, due to the difficulty in obtaining the latter for research purposes. Despite the fact that we raised the number of teeth in the sample to three to imitate the oral scenario, the retainer is still short in contrast to the oral situation, which is regarded as a restriction for this study. Because this was an in vitro investigation, caution should be used when extrapolating the findings to clinical applications. Multiple variables influence the intraoral environment, including saliva, oral habits, and food.

## Conclusions

The following conclusions were reached while taking into account the limitations of this in vitro study:

- During the ageing process, the fixed lingual retainers in all groups did not fail due to thermocycling or loading cycling. GO TO paste and Assure Plus Resin bonding produced various results for each of the investigated groups. However, all of the evaluated retainers in all groups offered strong enough bonding for use in clinical settings.
- In comparison to all studies retainers evaluated, CAD/CAM fiber-reinforced composites (FRCs) had the highest debonding force values, while CAD/CAM polyether ether ketone (PEEK) had the lowest.
- Bond-A-Braid™ had the highest connector retainer displacement values of any of the retainers evaluated, whereas CAD/CAM PEEK had the lowest.
- The CAD/CAM FRCs and PEEK that are more fitting and simpler to bond using conventional methods provided enough debonding force but only provided a small degree of deflection to preserve normal tooth function.
- The ARI score was significant because it should be taken into account when choosing an orthodontic adhesive. All adhesive left on the teeth were found to be the most prevalent in all related groups, indicate failure of the retainer’s composite interfaces.
- In this study, braided rectangular wire may be suggested for use as the preferred retainer since it showed adequate debonding force values, less ARI, and more connector retainer displacement that allowed tooth function to be normalized versus the CAD/CAM PEEK and FRC retainers.

## Data Availability

The datasets used during the current study are available from the corresponding author on reasonable request. All data analyzed during this study are included in this published article in the form of tables and figures.

## List of abbreviations

PEEK: Polyether ether ketone
FRC: Fiber reinforcement composite
ARI: Adhesive remnant index
N: Newton
